# Effects of bronchoalveolar lavage on Mycoplasma Pneumoniae pneumonia: A propensity score matched-cohort study

**DOI:** 10.3389/fped.2022.1066640

**Published:** 2023-01-04

**Authors:** Jinmiao Lu, Junqi Zhang, Guangfei Wang, Xiaobo Zhang, Zhiping Li

**Affiliations:** ^1^ Department of Pharmacy, National Children's Medical Center, Children's Hospital of Fudan University, Shanghai, China; ^2^ Department of Respiratory Disease, National Children's Medical Center, Children's Hospital of Fudan University, Shanghai, China

**Keywords:** mycoplasma, pneumonia, medication, bronchoalveolar lavage, child, infection

## Abstract

**Background:**

The purpose of this study was to evaluate the efficacy and safety of BAL in treating MPP.

**Methods:**

From January 2013 to January 2019, 1,689 pediatric patients with MPP were analyzed retrospectively. Patients were subdivided into BAL group and non-BAL group according to whether they received BAL treatment within seven days after admission. The propensity score matching method matched patients' baseline characteristics (1:1). The primary outcomes were hospital stays and the cure rate. Secondary outcomes included mortality, co-infection, repeat hospitalization within 30 days, and total cost of treatment.

**Results:**

After matching, 524 patients (BAL: 262; control: 262) were recorded. The BAL group had significantly shorter hospital stays (OR: 0.5, 95% CI: 0.4–0.7). Meanwhile, BAL did not significantly modify the cost, co-infection rate, and mortality. In subgroup analyses, the group with BAL intervention within three days had a significantly shorter hospital stay (OR: 0.4, 95% CI: 0.3–0.5) compared with the group with BAL intervention three days after admission.

**Conclusions:**

Early BAL intervention is a better treatment than conventional drug therapy alone, and no significant complications were seen in this study. BAL intervention has an excellent clinical benefit. The earlier the intervention, the better the effect.

## Introduction

Pneumonia is the third leading cause of death worldwide (1). More than 40% of community-acquired pneumonia (CAP) cases are due to Mycoplasma pneumoniae pneumonia (MPP), and 18% of children with MPP require hospitalization (2, 3). In the United States, one-quarter of hospitalized children with pneumonia older than five years are MPP infections (4). Mycoplasma has no cell wall, and Macrolide drugs, rather than penicillin or cephalosporin, are the preferred antidote (5, 6). Resistance to Macrolide drugs has been increasing worldwide, particularly in Asia, which amounts to 90%–100% (7, 8). Refractory MPP (RMPP) is a condition that does not improve after seven days of standard Macrolide therapy (9). In recent years, the increasing proportion of RMPP has posed a significant challenge to paediatricians (10). A South Korean study of 5,294 children with pneumonia showed RMPP rates of more than 20% (11). Patients with RMPP must be treated with tetracycline or fluoroquinolones (12, 13). Tigecycline can also be added when patients do not respond to fluoroquinolones (14). However, such multi-drug therapies may disrupt the gut microbial balance (15) and increase the risk of adverse drug reactions (16). In particular, these new drugs may be traced to life-long sequelae in children, including tetracycline teeth, cartilage dysplasia, etc (17).

Bronchoalveolar lavage (BAL) can shorten the course of pneumonia and improve the prognosis in neonates with severe pneumonia (18, 19). In combination with medication, BAL gets a better treatment than medication alone in children with RMPP (20). Clinical studies have shown that BAL can improve the recovery of lung injury in MPP patients with multipolar consolidation (21). The improvement of BAL in the initial stage of MPP is still unknown. This study was designed to evaluate BAL's efficacy in treating children with MPP from 2013 to 2019 at the National Children's Medical Center, in Shanghai, China.

## Methods

### Patients source

All the children in this cohort were infected with Mycoplasma pneumoniae pneumonia. From January 2013 to January 2019, the patients were admitted to a tertiary hospital with 1,689 pediatric beds in Shanghai, China. The IgM of mycoplasma pneumonia was more than 1: 160 in all pediatric patients. Or all were positive for mycoplasma by throat swab rapid culture results/DNA assay. With the spread of BAL technology, the number of patients admitted to the hospital between January 2013 and January 2019 who received early BAL active intervention has been increasing year by year, thus forming a treatment group. **S**ome patients did not receive BAL active therapy and still received conventional drug therapy, so they created a control group. The exclusion criteria included: (1) severe pneumonia in patients with viral, bacterial, chlamydial, and other pathogens; (2) the patient had a history of chronic respiratory diseases such as asthma bronchitis, obstructive bronchitis, (3) congenital diseases such as immune system defect and severe hypofunction of heart, (4) a history of allergy to Macrolide drugs. The study protocol was subject to approval by the Institutional Review Board of the Pediatric Hospital affiliated with Fudan University (FELS2014–084). Due to the observational nature of the retrospective study, written informed consent was waived.

### Treatment methods and evaluation

In this study, the patient's baseline characteristics, treatment implementation and clinical outcomes were collected by reviewing medical records. All the children received standard 7-day Macrolide treatment after admission: Azithromycin injection (Pfizer), IV, at a dose of 10 mg/kg d, once daily. The course of treatment is not greater than seven days.

In the BAL group, the parents of the children needed to consent to bronchoalveolar lavage, and the family members of the children signed the informed consent form. The contraindications of BAL in children included: (1) severe hypofunction of heart and lung; (2) severe arrhythmia: atrial, ventricular fibrillation and flutter, atrioventricular block; (3) high fever, (4) severe arrhythmia Severe hemorrhagic disease, Coagulopathy, severe pulmonary hypertension and possible massive hemoptysis; (5) severe malnutrition. The procedure of BAL was as follows: routine preoperative preparation, monitoring of vital signs, oxygen inhalation by face mask, local anaesthesia with 2% lidocaine, feeding fiberoptic bronchoscope from nasal cavity; Combined with preoperative imaging examination, the location of the lesion was selected, the distal end of the bronchoscope was fixed, and 37°C, 0.9% sodium chloride solution was injected into the Bronchus for bronchoalveolar lavage. 5–10 ml of lavage fluid was aspirated at negative pressure and repeated 2–4 times.

All the patients were assessed on discharge and were classified into four grades: cured, effective, ineffective, and dead, according to clinical manifestation, laboratory and imaging examination. Total recovery: the child's body temperature returned to normal, with no noticeable cough, shortness of breath or other clinical symptoms; inflammatory indicators were usual, and imaging examination suggested that the absorption of inflammatory shadow; effective: the child's body temperature is average, respiratory symptoms better than before; ineffective: the results showed that the clinical manifestation, laboratory examination and imaging examination of the patients were not improved or even worsened. The cure rate = [(number of cured cases + number of effective cases)/total number of cases] × 100%.

### Statistical approach

We used the statistical package STATA15.0 as the statistical analysis software. Continuous variables between the two groups were expressed as mean ± standard deviation. Category variables are reported in percentages. Comparisons were made concerning constant variables using the Mann-Whitney *U* or Student's t-test and category variables using the chi-square test. *P *< 0.05 indicated that the difference was statistically significant. The Kaplan-Meier method was used to evaluate the total length of hospital stay in both groups, and odds ratios (OR) and 95% confidence intervals (CI) were calculated.

Two groups of subjects were matched 1:1 nearest neighbour using propensity score matching (PSM), with the calliper value set to 0.2. The variables for generating propensity score included age, sex, race, Comorbidities, d-dimer, and CRP level. The standard difference was utilized to evaluate the balance between the two groups. The difference of < 0.1 indicated that the balance of the variables between the two groups was better. In addition, subgroup analysis was performed to analyze the outcomes of BAL patients at different times of the intervention.

We chose six variables for PSM matching for three reasons. First, a previous study of the same type screened for five parameters, including age, sex, race, c-reactive protein, and comorbidities (22). Second, a previous study showed a gender difference in treatment outcomes for pneumonia (23). Meanwhile, the immunopathogenesis of MPP is regarded as age-dependent (24). Age may be the sole predisposing factor of MPP (25). Third, elevated D-dimer levels can be used as a predictor of RMPP and complications (26, 27).

## Results

**Figure 1:**
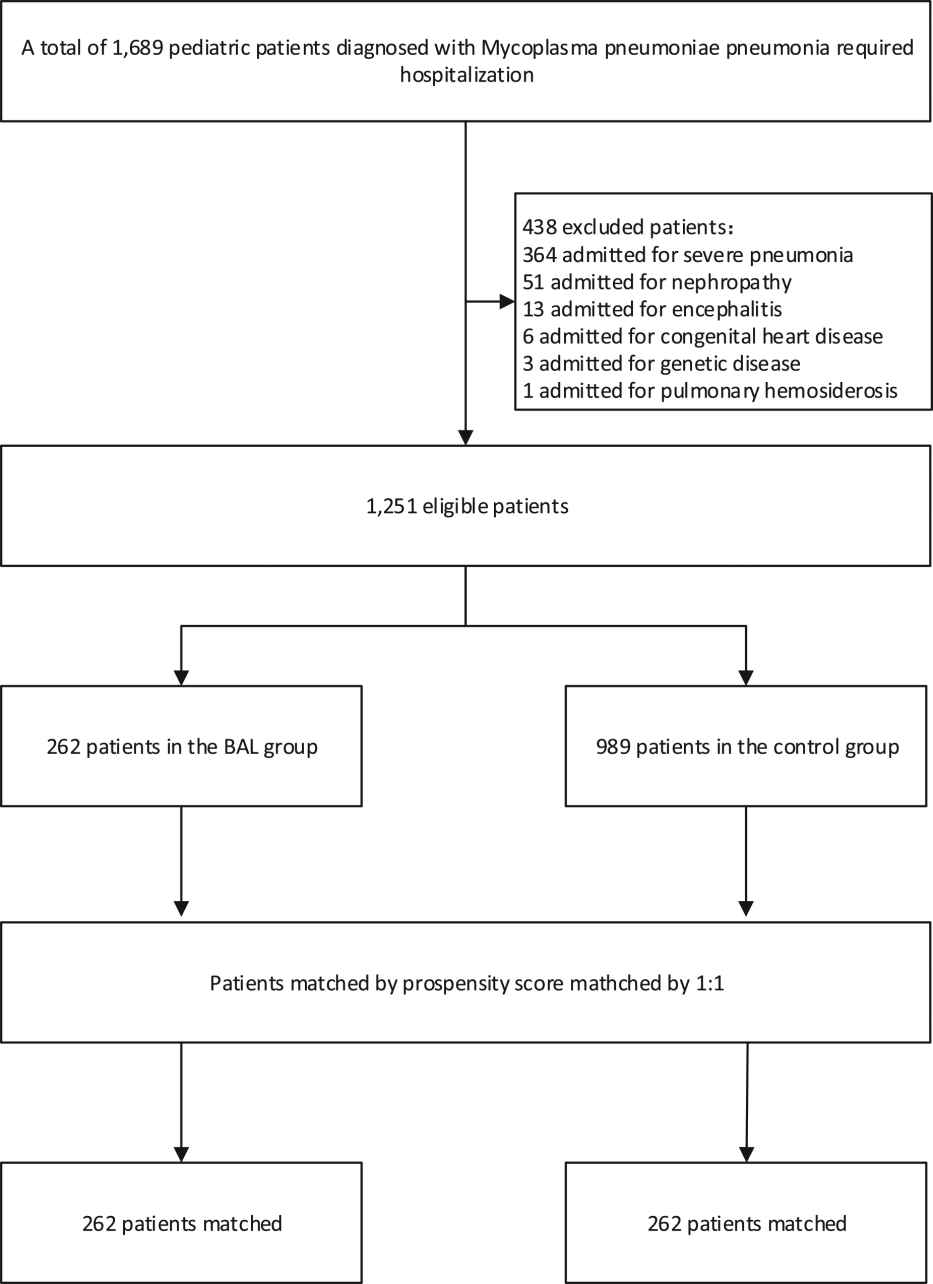
Patient flowchart.

There were 262 cases in the BAL group and 989 cases in the control group. After 1∶1 matching, 262 patients were included in the analysis (Figure 1). The baseline characteristics of pre-matched patients are shown in Table 1. There were significant differences between the two groups in CRP, pneumonia complications (alveolar fluid), and race type (*p* < 0.05). These significant differences may be due to a lower rate of pneumonia complications in the control group than in the BAL group (9.7% vs. 22.2%). After PSM, there were no statistically significant differences in all demographic information between the two groups (*p* > 0.05).

**Table 1:** Baseline characteristics before and after propensity score matching.

Demographic characteristics	Unmatched cohort			Matched cohort		
	BAL *n* = 262	Control *n* = 989	*P* value	BAL *n* = 262	Control *n* = 262	*P* value
**Age, mean (SD), y**	5.3 ± 2.7	5.6 ± 1.0	0.09	5.3 ± 2.7	5.3 ± 3.5	1.00
**Gender, Male (%)**	132 (50.4%)	503 (50.9%)	0.89	132 (50.4%)	140 (53.4%)	0.48
**CRP (ng/L)**	8.0 ± 0.1	11.0 ± 6.1	<0.001*	8.0 ± 0.1	8.0 ± 0.1	1.00
**D-dimer (mg/L)**	1.5 ± 1.7	1.3 ± 0.4	0.06	1.5 ± 1.7	1.6 ± 1.9	0.44
**Pulmonary complications, *n* (%)**	58 (22.2%)	96 (9.7%)	<0.001*	58 (22.2%)	63 (24.1%)	0.60
**Han Race, *n* (%)**	262 (100)	982 (99.3)	0.03*	262 (100)	262 (100)	1.00
**Lactic dehydrogenase (IU/L)**	362.7 ± 139.5	372.7 ± 131.4	0.28	362.7 ± 139.5	373.5 ± 113.6	0.33
**Leucocyte count, × 10^9^/L**	3.0 ± 2.0	2.9 ± 1.7	0.42	3.0 ± 2.0	3.1 ± 1.9	0.56
**Lymphocytes (%)**	36.4 ± 15.2	34.7 ± 15.1	0.11	36.4 ± 15.2	35.4 ± 14.3	0.44
**Neutrophil count, × 10^9^/L**	4.9 ± 2.9	4.9 ± 3.2	1.00	4.9 ± 2.9	5.0 ± 3.3	0.71
**Neutrophil (%)**	54.3 ± 17.6	55.6 ± 16.3	0.26	54.3 ± 17.6	56.8 ± 15.3	0.08
**Procalcitonin (ng/ml)**	0.1 ± 0.3	0.2 ± 1.1	0.15	0.1 ± 0.3	0.2 ± 0.7	0.07
**Neutrophil-Lymphocyte Ratio**	2.6 ± 2.7	2.4 ± 2.6	0.27	2.6 ± 2.7	2.4 ± 4.2	0.52
**Platelet, × 10^9^/L**	317.2 ± 137.9	347.9 ± 149.2	0.003*	317.2 ± 137.9	323.4 ± 161.3	0.64

* *p* < 0.05.

**Figure 2:**
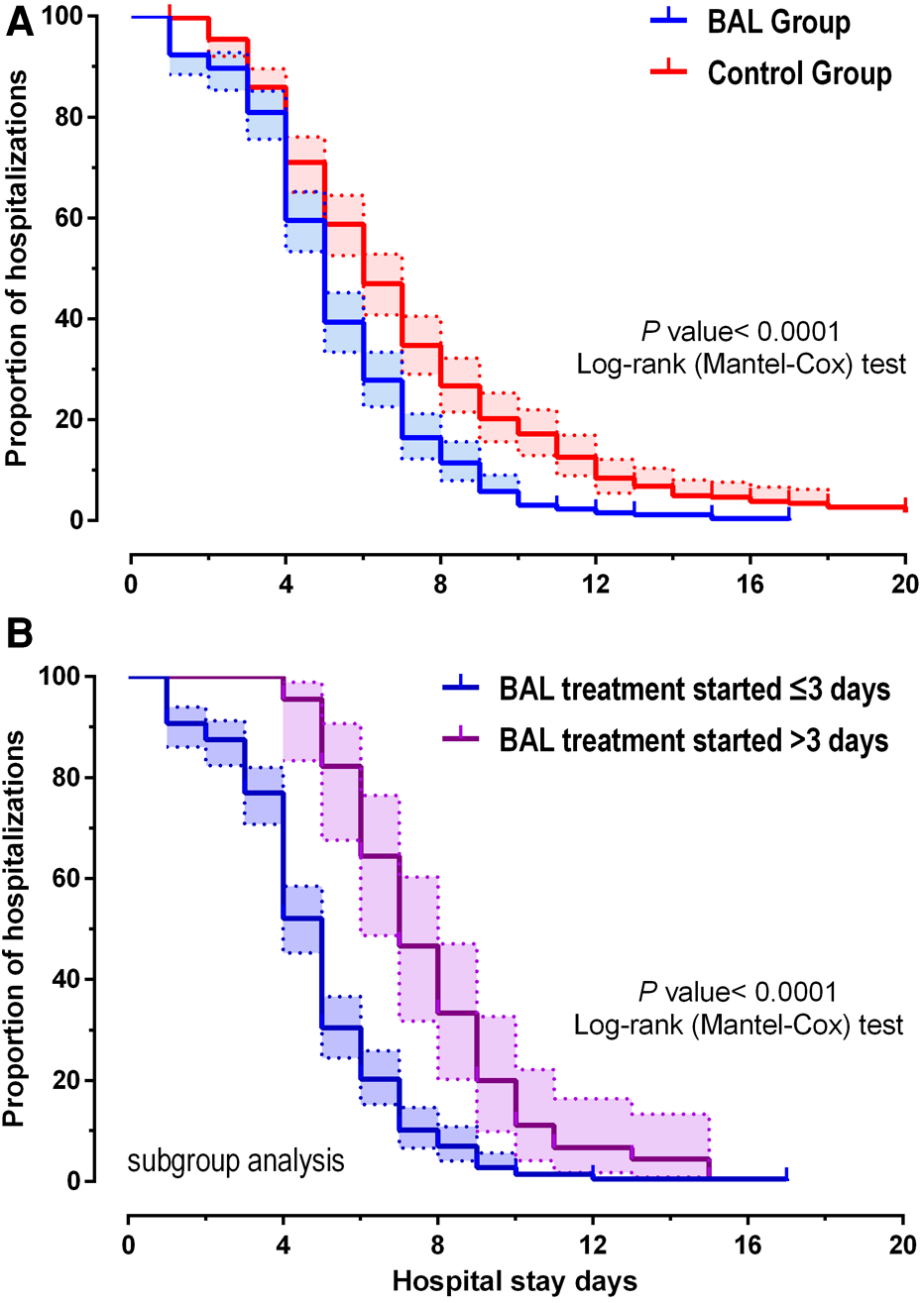
Kaplan-Meier plotter showing the discharge time for MMP patients (**A**). (**B**) shows the difference in length of stay between days 3 and 3 when the lavage was performed.

Clinical outcome results are shown (Table 2): among the 262 patients, the mean length of hospital stay was 5.3 days in the BAL group and 7.4 days in the control group. As shown in Figure 2A, using BAL reduced the size of hospital stay by 2.1 days compared with the control group (*p* < 0.001). And there was a significant difference in hospital length of stay between the two groups before and after matching. In the matched cohort, the considerable difference in the incidence of concurrent infection between the BAL group and the control group disappeared after seven days of treatment. There were no significant differences between the two groups in the rate of repeated hospitalization within 30 days, the rate of death, and the rate of complicated infection. In addition, we performed a subgroup analysis of the BAL group (Figure 2B). Subgroup analysis showed that BAL intervention within three days of hospitalization significantly decreased the time to final discharge compared with the group after three days (*p* < 0.001). Notably, we significantly reduced RMPP conversion rates in all BAL groups before and after matching (*p* < 0.001). Compared with the control group, the BAL group had significantly shorter hospital stays (OR: 0.5,95% CI: 0.4–0.7). Meanwhile, BAL did not substantially modify the total cost, co-infection rate, and mortality. In subgroup analyses, the group with BAL intervention within three days had a significantly shorter hospital stay (OR: 0.4,95% CI: 0.3–0.5) compared with the group with BAL intervention three days after admission.

**Table 2:** Outcomes in the original unmatched cohort and matched cohort.

**Outcomes**	**Unmatched cohort**		** **	**Matched cohort**		** **
	**BAL *n* = 262**	**Control *n* = 989**	***P* value**	**BAL *n* = 262**	**Control *n* = 262**	***P* value**
**Inpatient days, average ± SD**	5.3 ± 2.6	7.4 ± 6.0	0.001*	5.3 ± 2.6	7.4 ± 6.1	0.001*
**The cure rate (%)**	262 (100)	986 (99.7)	0.27	262 (100)	262 (100)	1.00
**Mortality, *n* (%)**	0 (0.0)	1 (0.1)	0.31	0 (0.0)	0 (0.0)	1.00
**RMPP, *n* (%)**	8 (3.1)	160 (16.2)	<0.001*	8 (3.1)	45 (17.2)	<0.001*
**Re-hospitalizations within 30 days, *n* (%)**	0 (0.0)	0 (0.0)	1.00	0 (0.0)	0 (0.0)	1.00
**Combined infection, *n* (%)**	1 (0.4)	35 (3.5)	0.007*	1 (0.4)	2 (0.8)	0.56
**Costs, RMB (yuan)**	10,268.2 ± 3675.2	10,632.5 ± 8337.5	0.49	10,268.2 ± 3675.2	10,581.7 ± 8648.3	0.59

* *p *< 0.05.

## Discussion

BAL can improve pulmonary imaging of RMPP patients and shorten hospitalization time (20, 28). However, some clinical studies have shown that BAL can significantly prolong the hospital stay and increase the total cost (21). Why the contradictory results? We think that the key factor is the timing of BAL initiation, a difference that has not been strictly distinguished in previous studies. Usually, the paediatrician must confirm the diagnosis of RMPP after seven days of ineffective Azithromycin therapy before starting BAL (29).

In contrast, our study takes the lead in adopting BAL in patients with early MPP rather than RMPP. The results showed that BAL intervention within seven days considerably shortened hospital stay, regardless of whether they were later RMPP patients. Moreover, subgroup analyses showed a better treatment benefit when BAL was performed within three days of admission than after three days (Figure 2B). All these suggest that the earlier BAL is used, the better clinical results will be achieved.

It is worth pointing out that BAL can also reduce the incidence of RMPP (MPP to RMPP conversion rate) by 5.5 times (3.1% vs. 17.2%, *p *< 0.001). We were amazed by this dramatic improvement, and we speculate that there may be three reasons. First, BAL could decrease the abundance of MP in the lung. MP can cause epithelial cell damage and reduce the clearance of mucociliary cells (30). The degree of epithelial injury directly resulted from MPP's severity (31). The abundance of MP in the lung was positively correlated with the length of hospital stay, peak fever and serum c-reactive protein level (32, 33). The high load of MP in the lungs of pediatric patients was significantly correlated with the severity of pneumonia (34, 35). Second, BAL can clear away mucus plugs, improve child ventilation, and promote the rapid absorption of imaging large patchy shadows. Studies have shown that 75% of children with RMPP have mucus plug formation (36), and children with mucus plug have longer radiology clearance, signing up to 8 weeks (22). Third, BAL can eliminate cytokines, lipids and other inflammatory substances. For example, the up-regulation of multiple cytokines in bronchoalveolar lavage fluid exacerbates disease severity (37, 38). Increased glycerophospholipids, sphingolipids, and fatty acyl groups in the lungs lead to alveolar and endothelial cell death (39, 40).

While we support the positive efficacy of early BAL interventions, we are also concerned about the increased cost and rate of co-infection associated with BAL. Fortunately, we found no significant change in total hospital costs and co-infection rate in the BAL group. There are two possible causes for this. First, BAL in Shanghai costs about $20 per session. In contrast, the same single operation in the United States costs at least $400 (41). Second, patients with RMPP usually need multiple BAL treatments, which may prolong the hospital stay and increase the cost of treatment. On the contrary, our study shows that if BAL intervention is performed in advance, most patients need only one lavage. Therefore, we conclude that BAL's timely intervention strategies are cost-effective in reducing hospital length of stay without increasing total costs.

There are certain inadequacies in this study. Despite the use of the PSM method in this trial, some other probable factors are not considered, which may be biased. As a single-centre study, the small sample size has some limitations. Therefore, a larger scale of multicenter controlled trials is needed to confirm the BAL effect in the future.

## Conclusion

Timely BAL treatment can postpone the progression of MPP and provide a larger therapeutic time window for treating MPP in children. BAL within seven days after hospitalization can shorten the healing time of MPP children and promote their recovery and early discharge. BAL intervention therapy had better efficacy, similar safety, and better clinical benefit. Therefore, early BAL can not only remove inflammatory substances but also reduce the abundance of mycoplasma in the lung and promote the recovery of pediatric patients.

## Data Availability

The raw data supporting the conclusions of this article will be made available by the authors, without undue reservation.
